# “Quantity-effect” research strategy for comparison of antioxidant activity and quality of *Rehmanniae Radix* and *Rehmannia Radix* Praeparata by on-line HPLC-UV-ABTS assay

**DOI:** 10.1186/s12906-019-2798-8

**Published:** 2020-01-17

**Authors:** Hong-Ying Li, Jiang-Ji Fang, Hua-Dan Shen, Xue-Qiong Zhang, Xiao-Ping Ding, Jun-Feng Liu

**Affiliations:** 1 Hubei Institute for Drug Control, Wuhan, 430075 Hubei China; 2 School of Chemistry, Chemical Engineering and Life Sciences, Wuhan University of Technology, Wuhan, 430070 Hubei China; 3 MOE Key Laboratory of Chinese Medicine Resource and Compound Prescription, Hubei University of Chinese Medicine, Wuhan, 430065 Hubei China

**Keywords:** HPLC-UV-ABTS, *Rehmanniae Radix*, *Rehmannia Radix* Praeparata, Quality evaluation, HPLC-FTMS

## Abstract

**Background:**

Quantitation analysis and chromatographic fingerprint of multi-components are frequently used to evaluate quality of herbal medicines but fail to reveal activity of the components. It is necessary to develop a rational approach of chromatography coupled with activity detection for quality assessment of herbal medicines.

**Methods:**

An on-line HPLC-ultraviolet detection-2,2′-azino-bis(3-ethylbenzothiazoline-6-sulfonic acid) free radical scavenging (HPLC-UV-ABTS) method was developed to obtain the chromatographic fingerprints and ABTS^+•^ inhibition profiles (active fingerprints) of *Rehmanniae Radix* (Dihuang) and *Rehmannia Radix* Praeparata (Shu Dihuang). Eighteen compounds showing ABTS^+•^ inhibition activity were identified by HPLC-fourier-transform mass spectrometry (HPLC-FTMS). Verbascoside was used as a positive control to evaluate the total activities of the samples and the contribution rate of each compound. The similarities of the chromatographic and active fingerprints were estimated by the vectorial angle cosine method.

**Results:**

The results showed that the HPLC-UV-ABTS method could efficiently detect antioxidant activity of the herbal medicine samples. The antioxidants were different between the two herbs and several new antioxidants were identified in Shu Dihuang. A function equation was generated in terms of the negative peak area (x) and the concentrations of verbascoside (y, μg/mL), y = 2E-07 × ^4^ - 8E-05 × ^3^ + 0.0079 × ^2^ + 0.5755x + 1.4754, *R*^2^ = 1. Iridoid glycosides were identified as main antioxidants and showed their higher contributions to the total activity of the samples. The total contributions of the three main active components in the Dihuang and Shu Dihuang samples to the total activity, such as echinacoside, verbascoside and an unknown compound, were 39.2–58.1% and 55.9–69.4%, respectively. The potencies of the main active components in the Shu Dihuang samples were two to ten times those in the Dihuang samples. Similarity values for S12 in the chromatographic fingerprints and S03, S12 and P03 in the active fingerprints were less than 0.9. The three batches of samples might show their different quality with the other samples.

**Conclusions:**

The results suggested that the combination of “quantity-effect” research strategy and the HPLC-UV-ABTS analysis method could comprehensively evaluate the active components and quality of Dihuang and Shu Dhuang.

## Background

*Rehmannia glutinosa* Libosch, one of the most common and important medicinal plants in China, is recorded in Chinese medical classic ‘Shennong’s Herba’. *R. glutinosa* based on the different processing methods is classified into three categories, namely fresh roots, dried roots (Dihuang), and steamed roots (Shu Dihuang), and they are applied to treat different diseases clinically. Shu Dihuang is usually applied to in the traditional Chinese medicine formulas that can nourish the Yin deficiency of liver, kidney and heart [1–3]. Many clinical and experimental studies have reported that the root of *R. glutinosa* possesses hypoglycaemic [4], anti-oxidant [5, 6] anti-cancer [7], anti-inflammatory [8], and immune-enhancement effects [9].

Iridoid glycosides are considered the main active ingredients in Dihuang. At present, a number of chemical constituents from Dihuang, such as iridoids, ionone glucosides, sesquiterpenes and phenylethanoid glycosides [10–12], have been isolated and identified. In the Chinese Pharmacopoeia (Ch. P), catalpol and verbascoside were quantified by HPLC methods to control the quality of Dihuang and Shu Dihuang, and 1,1-Diphenyl-2-picrylhydrazyl (DPPH) scavenging method was applied to distinguish verbascoside through thin layer chromatography (TLC) [13]. However, these methods might not be able to reveal efficiently the bioactivity of the compounds in Dihuang and Shu Dihuang.

Free radicals may be involved in cancer, ageing and cardiovascular diseases [14]. There has been a large research focus on antioxidants, especially plant-derived antioxidants. In order to identify efficiently antioxidants in complex extracts, the combinational techniques such as HPLC-DAD-DPPH, HPLC-DAD-ABTS, DPPH-CE-DAD and ABTS-CE-DAD for the analysis of DPPH^•^ and ABTS^+•^ scavenging activities, have been developed to screen radical scavengers in herbal medicines [15–19]. In these on-line methods, antioxidants separated by HPLC and CE will produce their negative peaks in real time detected at 517 nm for DPPH^•^ or at 414/734 nm for ABTS^+•^. This avoids the long and tedious process of bioassay-guided fractionation and isolation for structure elucidation in searching antioxidants from complex matrixes. Although the on-line methods have been used to evaluate the antioxidant activity of some herbal medicines, their effectiveness and extensive application need to be further verified.

In this study, an on-line HPLC-UV-ABTS method was developed to screen antioxidants in Dihuang and Shu Dihuang. The chromatographic and active fingerprints could be simultaneously obtained, and antioxidants from the two herbs were compared and identified by HPLC-FTMS. The angle cosine method was used to analyze the similarities of the chromatographic and active fingerprints. Thus, the quality of the herbs could be assessed comprehensively through the proposed research methods.

## Methods

### Materials and reagents

The information of the Dihuang and Shu Dihuang samples was listed in Additional file 1. Thirteen batches of Dihuang and 13 batches of Shu Dihuang samples were purchased from eleven manufacturers in China. Seven batches of Dihuang samples from the five manufacturers in Hubei province were recorded as S01, S02, S03, S04, S05, S06 and S12. Four batches of Dihuang samples from the four manufacturers in Anhui province were labeled as S07, S08, S09 and S13, while S10 and S11 were collected from the manufacturers of Guangzhou and Hangzhou, respectively. The thirteen of batches of Shu Dihuang samples from the same manufacturers were labeled as P01–13. Verbascoside was purchased from Chromadex (Irvine, America). ABTS was purchased from TCI (Shanghai, China).

HPLC and MS grade acetonitrile were obtained from Merck Drugs & Biotechnology (Darmstadt, Germany). Formic acid (FA) was purchased from Aladdin industrial corporation (Shanghai, China). Sodium chloride (NaCl), sodium dihydrogen phosphate (NaH_2_PO_4_), potassium chloride (KCl), potassium persulfate (K_2_S_2_O_8_), disodium hydrogen phosphate (Na_2_HPO_4_), sodium dihydrogen phosphate (NaH_2_PO_4_), sodium phosphate (Na_3_PO_3_) and acetic acid were analytical reagent grade. The water used was purified from a Millipore water purification system (Millipore, Bedford, MA, USA).

### Preparation of sample and standard solutions

All of the Dihuang and Shu Dihuang samples were dried at 60 °C under reduced pressure and pulverized to coarse powder. Sample powder (3 g) was extracted twice with 70 mL methanol for 40 min by ultra-sonication at room temperature. The extract solutions were filtered and mixed, then evaporated to dryness under vacuum and diluted with 15% ethanol to 10 mL. After being centrifuged at 10000 rpm for 10 min, an aliquot of 20 μL solution was analyzed by HPLC.

Verbascoside (7.78 mg and 3.81 mg) was weighed accurately and dissolved in 50 mL by methanol as the stock solutions. The different concentrations solutions, such as 3.05、6.10、18.7、50.3、99.6 and 155.6 μg/mL, were obtained through diluting the stock solutions with methanol. The standard solutions were used to set up the correlative graphs and equations between inhibiting peak areas and the concentrations of verbascoside.

### Preparation of ABTS solution

A stock solution was prepared with ABTS (0.110 g) dissolved in 100 mL of PBS solution (4.1 g NaCl, 0.135 g NaH_2_PO_4_, 0.7 g Na_2_HPO_4_ and 0.075 g KCl in 500 mL) containing 0.3 mM K_2_S_2_O_8_. A 2.0 mM stock solution was diluted using PBS solution containing 10% methanol to 0.3, 0.4, 0.5, 0.6, 0.7 and 0.8 mM, respectively. In on-line analysis, ABTS solution was freshly prepared and protected from light and cooled in an ice bath.

### HPLC-UV-ABTS assay

The HPLC-UV-ABTS system was reported in our previous publication [20]. The on-line instrumentation consisted of a Waters HPLC for chromatographic fingerprint analysis at 334 nm or 250 nm and an Ultimate 3000 UV detector for ABTS^+•^ scavenging analysis at 734 nm. ABTS solution was delivered with an injection pump of Pickering PCX Della (Pichering Laboratories Inc., USA) at the flow rate of 0.5 mL/min after PDA detector, and then the elution was mixed with ABTS solution after PDA detector through a reaction coil with 1.4 mL volume. The reaction products were determined at 734 nm by UV detector (Ultimate 3000). All other parts of the HPLC-UV-ABTS system were interconnected using polyether ether ketone (PEEK, 0.5 mm i.d.) tubes and T-shaped PEEK tubes.

Agilent Extend C_18_ columns (250 mm × 4.6 mm, 5 μm) were used for all chromatographic separations. The mobile phase comprised 0.1% (v/v) acetic acid (A) and acetonitrile (B). Gradient elution was performed as follows: 2–4% B in 0–10 min, 4–15% B in 10–40 min, 15–25% B in 40-55 min, 25–30% B in 55-60 min and 30–95% B in 60-65 min. The flow rate was 1.0 mL/min and column temperature was maintained at 30 °C. The detection wavelength was set at 334 and 250 nm for acquiring chromatograms. An aliquot of 20 μL sample solution was injected for HPLC-UV-ABTS analysis.

### HPLC-FTMS analysis

Thermo Scientific Orbitrap Fusion Tribrid HPLC-MS system was controlled by Xcalibur software (Version 3.0). The chromatographic conditions were the same as those in the HPLC-UV-ABTS analysis. The mobile phase was split after the DAD detector by a T-tube connected using two PEEK tubes with the same inner i.d. and different length. The split solution with a flow rate of 0.2 mL/min finally arrived at the MS detector.

The MS experiments were performed to get an accurate MS and MS^2^ of the new analogue. The ionization source was operated in the negative ionization modes with the flow rates of the sheath gas and auxiliary gas at 40 and 10 arbitrary unit, respectively, capillary temperature at 320 °C, ion spray source capillary at 2.5 kV, source current at 100 μA. Six scan events were selected in the MS experiment. Scan event 1 was used for full scan with scan range from 100 to 1000 m/z and resolution 60,000. Scan events 2–6 were used to produce MS^2^ through dependent scans selecting the 1th to 5th most intense ions in scan event 1 and resolution 15,000. Collision energy was set at 35 V using High Energy Collision Dissociation (HCD).

### Similarity of the two-dimensional fingerprint

It is well known that the samples with similar chromatographic fingerprint possess likely similar properties. Therefore, the consistency of herbal medicines can be tested through comparing the similarity between the chromatographic fingerprints of samples and the reference/standard fingerprints.

In this paper, a data analysis method was employed to evaluate the similarity of the two-dimensional fingerprint [21]. The chromatographic and active fingerprints from HPLC-UV-ABTS method were represented mathematically by a vector of their chromatographic peak areas and inhibiting peak areas. Thus, taking the two-dimensional fingerprint as an example, assume that vector *X* (*x*_1_, *x*_2_, *x*_3_, …, *x*_n_) (| *X* | = $\sqrt{{x_1}^2+{x_2}^2+{x_3}^2+\cdots \cdots {x_n}^2}$) represents the chromatographic or active fingerprint and the other vector *Y* (*y*_1_, *y*_2_, *y*_3_,…, *y*_n_) (| *Y* | = $\sqrt{{y_1}^2+{y_2}^2+{y_3}^2+\cdots \cdots {y_n}^2}$) represents the reference fingerprints, while the vector angle of *X* and *Y* is calculated by the formula. The two vectors are more similar when the cosine values are near 1.

$$ \cos \theta =\frac{X\cdot Y}{\mid X\mid \times \mid Y\mid } $$

Where *x*_i_ denotes absolute peak area of chromatographic or active fingerprints, and *y*_i_ denotes mean area of the peak.

## Results

### Optimization of HPLC-UV-ABTS analysis conditions

HPLC-UV-ABTS was developed for the determination of free radical scavengers in the complex matrixes due to a relatively simple and stable instrument system. Methanol/acetonitrile-acid aqueous solution used as mobile phases were compatible with the on-line detection of ABTS^+•^ inhibition after column or UV detector. Baseline was relatively stable due to the buffer effect of ABTS dissolved in buffer solutions to mobile phases.

The concentrations of the ABTS solution affected directly the sensitivity of the HPLC-UV-ABTS method. In view of this, the different concentrations of ABTS including 0.3, 0.4, 0.5, 0.6, 0.7 and 0.8 mM were investigated. The results showed that the stronger inhibition peaks were produced when the lower concentrations of ABTS were selected, while the higher concentrations induced strong baseline draft. Finally, 0.6 mM ABTS was used to analysis the antioxidants in the two herbs. Additionally, the flow rates at 0.25 and 0.5 mL/min for the ABTS solution were optimized. The flow rate of 0.5 mL/min was finally selected owing to a long time delay from the lower flow rate for ABTS^+•^ scavenging analysis.

### Method validation for HPLC-UV-ABTS

The analytical methods of the chromatographic and active fingerprints were validated based on retention times, peak areas and ABTS^+•^ inhibition peak areas. The intra-day precisions of HPLC-PDA were 0.086–0.75% (*n* = 5) for retention times and 0.70–1.46% (*n* = 5) for peak areas, while the inter-day precisions were 0.10–0.83% (*n* = 5) for retention times and 1.18–3.37% (*n* = 5) for peak areas. The intra-day precisions of HPLC-UV-ABTS were within the range of 0.028–0.93% (*n* = 5) for retention times and 0.069–0.95% (*n* = 5) for negative peak areas, whereas the inter-day precisions were 1.23–4.73% (*n* = 5) for retention times and 1.66–5.71% (*n* = 5) for negative peak areas.

### On-line HPLC-UV-ABTS analysis

**Fig. 1:**
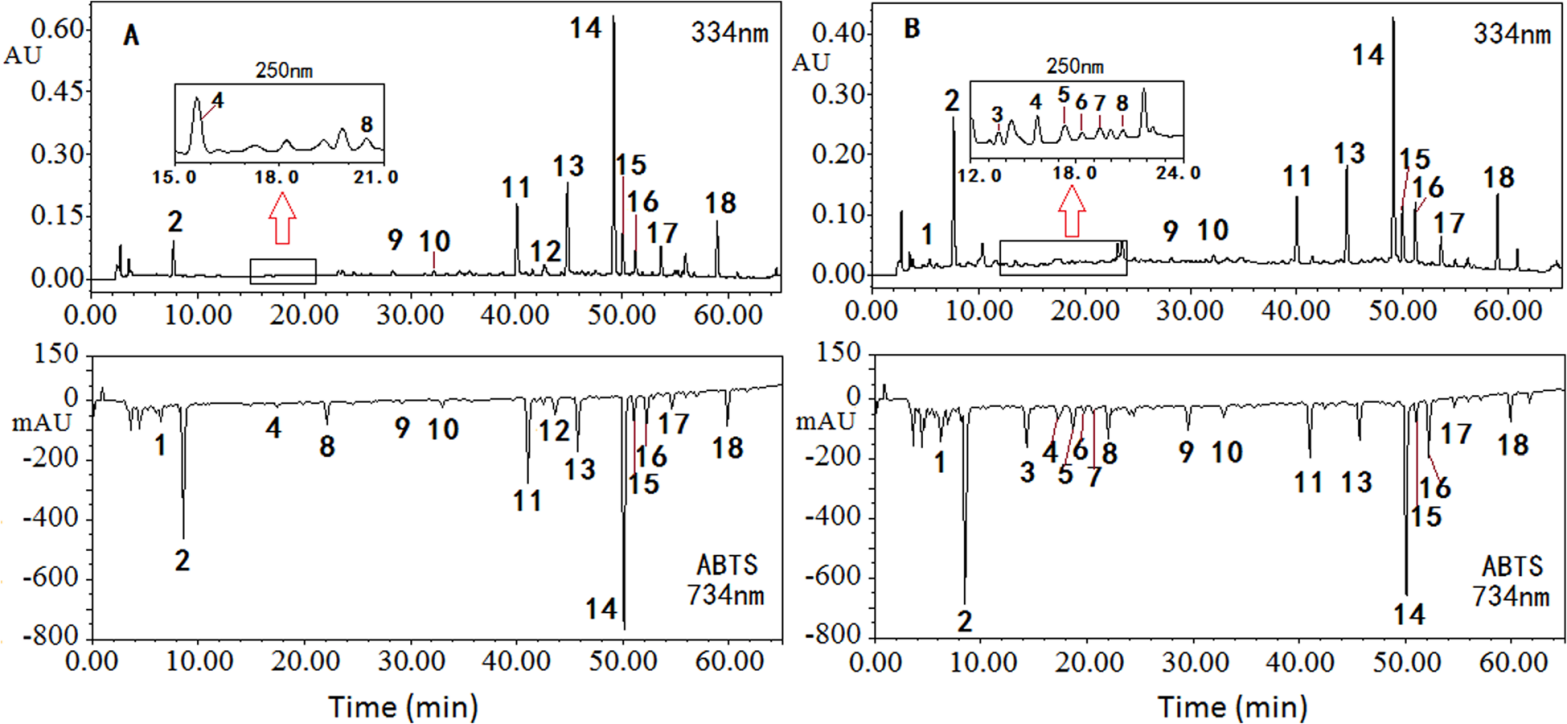
The chromatographic fingerprints and ABTS^+•^ inhibition profiles of Dihaung S07 (**a**) and Shu Dihuang P07 (**b**) at 334 nm and the partial chromatograms of the two samples at 250 nm. A partial chromatogram at 250 nm from 15.0 min to 21.0 min in chromatogram of Dihuang, a partial chromatogram at 250 nm from 12.0 min to 24.0 min in chromatogram of Shu Dihuang

The chromatographic and active fingerprints of Dihuang and Shu Dihuang were shown in Fig. 1. Although chromatographic fingerprints of Dihuang and Shu Dihuang were similar, their active fingerprints were different. Fourteen negative peaks were observed in Dihuang (Fig. 1a), while there were 17 inhibition peaks in Shu Dihuang (Fig. 1b). The negative peaks 1, 2, 3, 4, 8, 9, 10 and 16 in Shu Dihuang were stronger than those in Dihuang. In addition, several new negative peaks were found in Shu Dihuang such as 5, 6 and 7, and negative peak 12 in Dihuang disappeared.

In Fig. 1a and b, few peaks were observed from 10 min to 40 min in the chromatographic fingerprints of the two herbs detected at 334 nm, while some weak peaks could be found at 250 nm and these peaks in Shu Dihuang showed obvious ABTS^+•^ inhibition activity, such as peaks 3–9. In view of stronger chromatographic signal at 334 nm for iridoid glycosides as main components in the two herbs, the chromatographic fingerprints were detected at this wavelength. Furthermore, ABTS^+•^ scavenging activity was detected usually at 414 nm and 734 nm, but the wavelength at 734 nm was selected in order to avoid the interference from the herb extracts at 414 nm.

### The activity evaluation of antioxidants in samples

As shown in Fig. 1, there were 18 antioxidants in the Dihuang and Shu Dihuang samples and their capacities for scavenging ABTS^+•^ were obviously different. However, it was difficult to evaluate and compare the activity of the antioxidants due to the impossibility of all the standards obtained. Some publications reported that the relative potencies of the antioxidants in complex extracts could be evaluated by the “quantity-effect” equation of a positive control [22, 23]. Thus the potencies of the antioxidants against free radical could be compared and antioxidant activity of a complex extract could be obtained through calculating the total potency of all the antioxidant components.

**Fig. 2:**
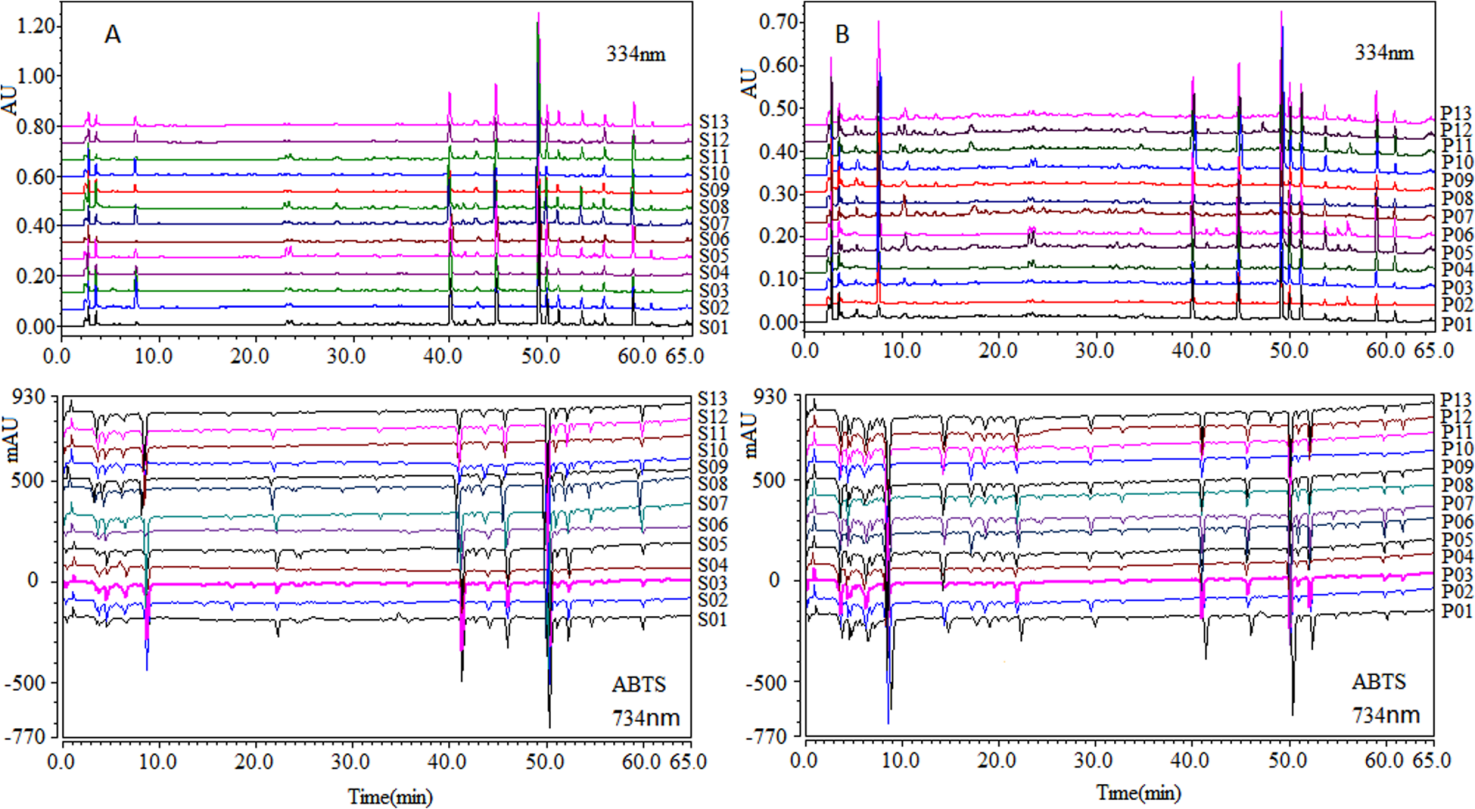
The chromatographic fingerprints and ABTS^+•^ inhibition profiles of 13 batches of Dihuang and 13 batches of Shu Dihuang samples. **a** Dihuang, **b** Shu Dihuang. The chromatographic fingerprints detected at 334 nm. ABTS+• inhibition profiles detected at 734 nm

**Fig. 3:**
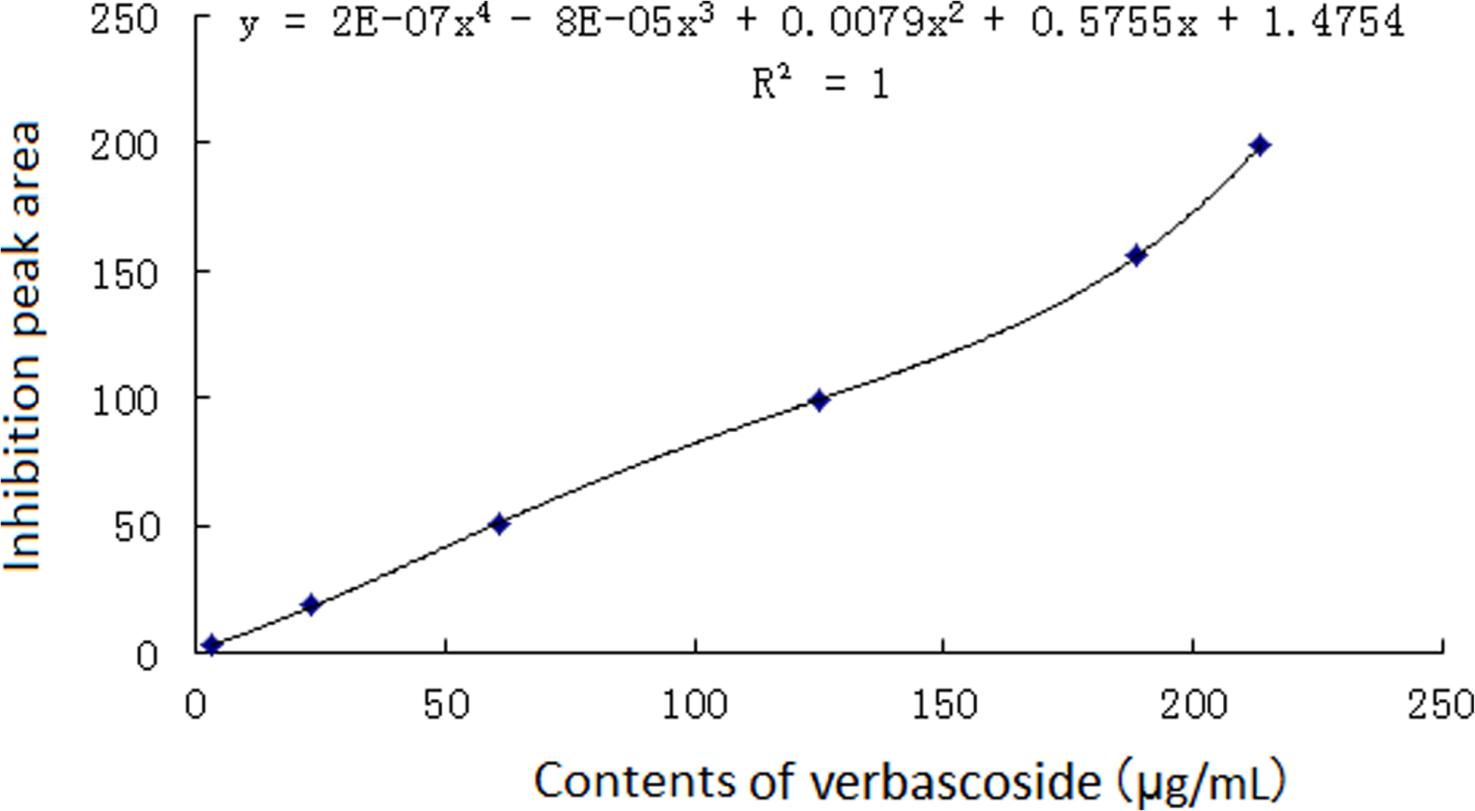
The correlative graphs and equations between the negative peak areas and concentrations for verbascoside against ABTS^+•^

In Figs. 1 and 2, verbascoside showed strong ABTS^+•^ scavenging activity in all the Dihuang and Shu Dihuang samples, so it was applied as a positive control. The functional equation of verbascoside y = 2E-07 × ^4^ - 8E-05 × ^3^ + 0.0079 × ^2^ + 0.5755x + 1.4754 (*R*^2^ = 1) was generated using negative peak areas (x) and C (y) (Fig. 3) (Additional file 2). If verbascoside (1 μg/ml) was presumed as a potency unit, the potencies of 18 antioxidants in the samples could be calculated by the functional equation (Additional file 3).

**Fig. 4:**
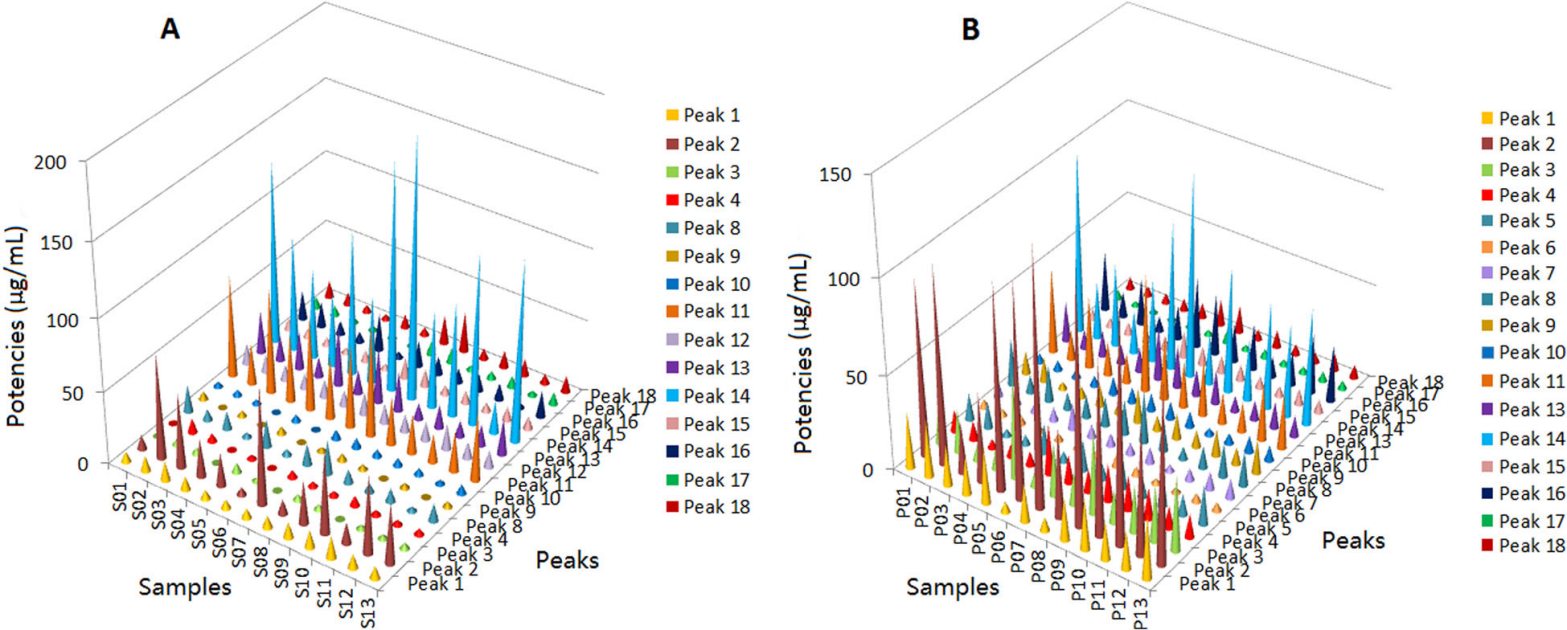
The potencies of the chromatographic peaks in the Dihuang and Shu Dihuang samples. **a** Dihuang, **b** Shu Dihuang

The potencies of 18 antioxidants in all of the Dihuang samples and Shu Dihuang samples were shown in Fig. 4a and b. The potencies of the antioxidants in Dihuang and Shu Dihuang were markedly different. In Fig. 4a, peak 2, 11, 12, 13 and 14 in the Dihuang samples showed strong ABTS^+•^ inhibition activity, while the antioxidant potencies of peak 1, 2, 3, 4, 11, 13, 14 and 16 in the Shu Dihuang samples were twice to ten times than those in the Dihuang samples (Fig. 4b). In addition, the activity of peak 1, 2, 3, 4, 8 and 16 in Shu Dihuang increased and this might attributed to the processing action of Dihuang. In our previous study, the HPLC-UV-DPPH method was developed to compare DPPH^•^ scavengers in Dihuang and Shu Dihuang [24], and the results were consistent with the above the results of HPLC-UV-ABTS analysis.

### Main antioxidants in Dihuang and Shu Dihuang

The eighteen negative peaks in Fig. 1 were identified by HPLC-FTMS and comparison with reference standards and the data in related literatures [12], and the MS data were listed in Table 1. As shown in Table 1, the most common inhibition peaks were iridoid glycosides, such as peaks 6–18 except for peak 8. In the Dihuang samples, the stronger negative peaks 11, 12, 13, 14 and 16 were identified as echinacoside, isomer of echinacoside, jionoside A1/A2, verbascoside and isoverbascoside, respectively. While peak 1, 4, 8 and 9 showing stronger activity in the Shu Dihuang samples were identified as glutinoside, geniposidic acid, mussarnosidic acid, and syringic acid-4-O-α-*L*-rhamnoside, respectively. Peaks 2 and 3 needed to be further identified.

**Table 1:** Identification of active components in Dihuang and Shu Dihuang sample by HPLC-FTMS

Peak	RT	[M-H]-	Chemical formula	MS^2^	Identification
1	6.22	397.0238	C_15_H_23_O_10_Cl	379.1123,276.9031,252.2112	glutinoside
2	8.55	495.1254	C_26_H_23_O_10_	477.1181,379.1190, 217.0684, 199.0581	unknown
3	14.37	731.2100	C_29_H_33_O_14_N_9_	505.1492, 323.0935, 263.0734,221.0633	unknown
4	17.25	373.1073	C_16_H_22_O_10_	283.0792,211.0580,167.0687,123.0431	geniposidic acid
5	18.61	375.1246	C_16_H_24_O_10_	315.1030,255.0831,213.0735,169.0843, 125.0586	8-epiloganic acid
6	19.60	389.1168	C_17_H_26_O_10_	183.0634,165.0531,139.0377	loganin
7	20.55	461.1592	C_20_H_30_O_12_	315.1039,297.0937,161.0429,135.0429	decaffeoylacteoside
8	21.94	375.1246	C_16_H_24_O_10_	315.1021,213.0738,169.0843,151.0740	mussarnosidic acid
9	29.57	343.0262	C_15_H_20_O_9_	299.1118,284.0858,197.0424,182.0192	syringic acid-4-*O*-α-*L-*rhamnoside
10	32.87	475.1747	C_21_H_32_O_12_	329.1194,311.1093,143.0583,161.0429	darendoside B
11	41.02	785.2394	C_35_H_45_O_20_	623.2108,461.1603,161.0218	echinacoside
12	43.60	785.2394	C_35_H_45_O_20_	623.2108,461.1603,161.0218	isomer of echinacoside
13	45.74	799.2570	C_36_H_48_O_20_	623.2102,605.1965,461.1597,315.1040	jionoside A1/A2
14	50.14	623.1865	C_29_H_36_O_15_	477.1354,461.1599,443.1508,315.1039,179.0321,161.0218	verbascoside
15	50.98	813.2729	C_37_H_50_O_20_	637.2254,491.1701,193.0477,175.0373	jionoside B1/B2
16	52.17	623.1865	C_29_H_36_O_15_	461.1600,315.1040,251.0525,179.0322,161.0218	isoverbascoside
17	54.61	637.2048	C_30_H_38_O_15_	491.1492,475.1773,461.4599,443.1497	jionoside D/leucosceptoside A
18	59.89	651.2176	C_31_H_40_O_15_	505.1632 475.1754 193.0477	martynoside/isomer

**Fig. 5:**
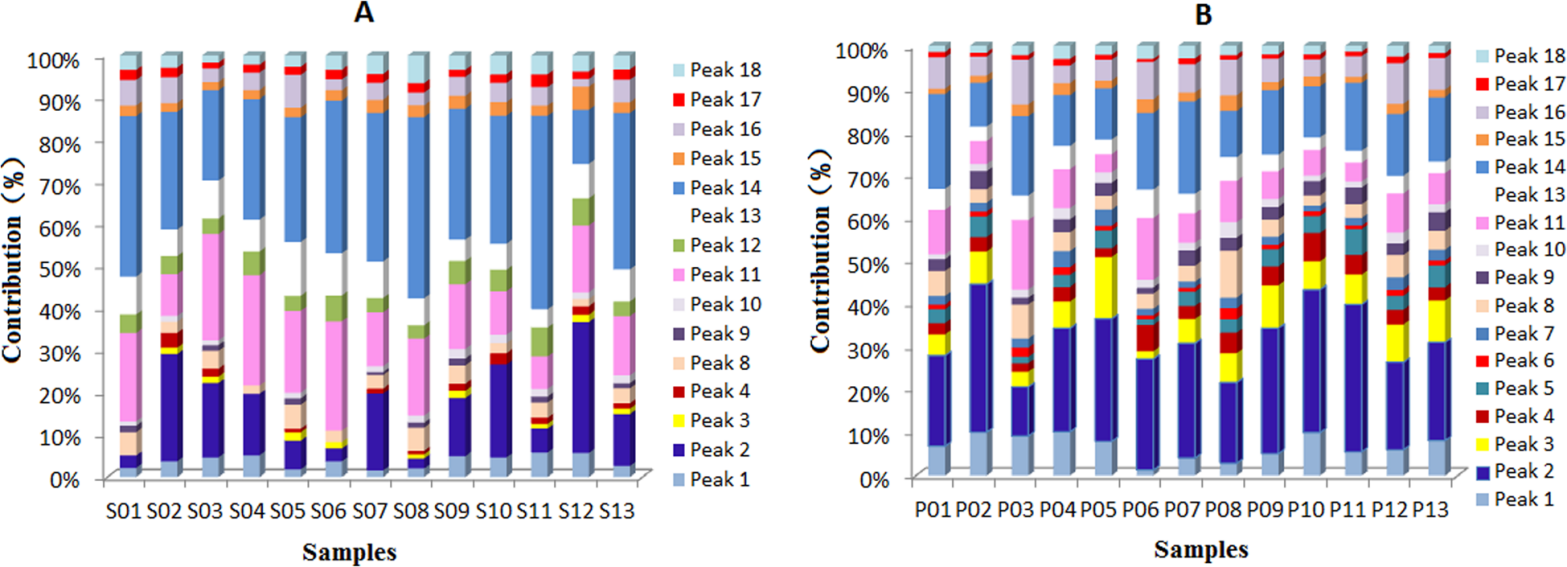
The contributions of the chromatographic peaks in the Dihuang and Shu Dihuang samples to the total activity. **a** Dihuang, **b** Shu Dihuang

Iridoid glycosides are main ingredients in Dihuang and Shu Dihuang, and verbascoside exhibiting stronger antioxidant activity is used to control quality of the two herbs in Ch. P. However, the activity of the other iridoid glycosides can’t be ignored. The contributions of 18 antioxidants in the Dihuang and Shu Dihuang samples to the total activity were shown in Fig. 5 (Additional file 4). In Fig. 5a, peaks 2, 11 and 14 in the Dihuang samples exhibited their high contributions, and the total contribution percentage of the three main active components in the Dihuang was 39.2–58.1%. Peaks 1, 2, 3, 11, 14 and 16 in the Shu Dihuang samples showed their high contributions (Fig. 5b), and the total contribution percentage of peak 2, 11 and 14 was 55.9–69.4%. In addition, the contributions of peak 1, 2, 3, 4, 8 and 16 in the Shu Dihuang samples were twice to twenty times than those in the Di Huang samples, while the contributions of peak 11, 13 and 14 in the Shu Dihuang samples decreased. The results indicated that the antioxidant activity of Dihuang might be changed after being processed.

### Similarity for Dihuang and Shu Dihuang samples

The chromatographic and active fingerprints of the thirteen batches of the Dihuang and Shu Dihuang samples from the different manufacturers were displayed in Fig. 2. As shown in Fig. 2a and b, the chromatographic and active fingerprints among the different samples were similar. In order to evaluate objectively the quality of the samples, the angle cosine method was employed to analyze the similarities of the two fingerprints. The angle cosine values of each chromatographic and active fingerprint to their respective reference chromatogram were calculated and listed in Table 2. The raw data were shown in Additional file 5 and Additional file 6. In Table 2, the different common peaks were used to evaluate the similarities of the two fingerprints. The peak 12 was not detected in the Shu Dihuang samples, so 8 common peaks were selected in the similarity evaluation of the chromatographic fingerprints. Fifteen and seventeen common peaks were used to analyze the similarity of the active fingerprints of the Dihuang and Shu Dihuang samples, respectively.

**Table 2:** Similarity of Dihuang and Shu Dihuang samples

Samples	Dihuang		Samples	Shu Dihuang	
	HPLC (9 common peaks)	ABTS (15 common peaks)		HPLC (8 common peaks)	ABTS (17 common peaks)
S01	0.994	0.982	P01	0.991	0.969
S02	0.954	0.957	P02	0.964	0.959
S03	0.903	0.898	P03	0.902	0.854
S04	0.952	0.958	P04	0.952	0.980
S05	0.988	0.978	P05	0.968	0.961
S06	0.975	0.969	P06	0.976	0.955
S07	0.994	0.974	P07	0.984	0.984
S08	0.989	0.974	P08	0.932	0.944
S09	0.992	0.988	P09	0.987	0.991
S10	0.991	0.934	P10	0.983	0.966
S11	0.941	0.946	P11	0.946	0.975
S12	0.840	0.716	P12	0.974	0.984
S13	0.994	0.993	P13	0.993	0.989

As shown in Table 2, the chromatographic and active fingerprints of the samples represented their differences to some extent. The similarity values of S03 and S12 in the two fingerprints were lower than those of the other samples, and their similarity values in the active fingerprints were less than 0.9. Moreover, the similarity value of P03 in the chromatographic fingerprints was lower than that of the other samples and less than 0.9 in the active fingerprints. Although the similarity values of the two fingerprints were differences, the quality of the Dihuang and the Shu Dihuang samples were basically consistent except for the several samples.

## Discussion

In this study, the HPLC-UV-ABTS method was developed to evaluate the main antioxidants and quality of the two herbs. Sensitivity of the on-line method correlated mainly concentration and flow rate of ABTS solution. Lower concentrations of ABTS solution were used, and stronger inhibition peaks in the samples could be obtained. Considering strong baseline drift induced by the higher concentrations of ABTS solution, 0.6 mM within the range of 0.3–0.8 mM was selected for the on-line analysis. Moreover, flow rate of ABTS solution affected directly the reaction times of the compounds and ABTS^+•^ when volume of reaction coil (1.4 mL) and flow rate of HPLC elution were stable. Flow rate of 0.5 mL/min for ABTS solution was applied due to a long time delay and lower resolution of the active fingerprints from the lower flow rates. Under the above analysis conditions, the method validation of HPLC-UV-ABTS was investigated through the intra-day and inter-day precisions of the chromatographic peaks, the negative peaks and retention times. The results indicated that the on-line method was stable and reproducible.

The chromatographic fingerprints of all the samples were detected at 334 nm, while some minor peaks were found in the chromatographic fingerprints of Shu Dihuang at 250 nm and showed their ABTS^+•^ scavenging activity. These minor peaks are hardly focused on owing to their weak signals in the chromatographic fingerprints. Chromatographic fingerprint mainly showing characteristics of strong peaks gave too much weight to major peaks than to minor peaks. In view of this, the similarities of the chromatographic and active fingerprints were evaluated and compared by the vector angle cosine method. Similarity values of several samples were less than 0.9. This might imply their different quality with the other samples. Thus, the combination method of the chromatographic and active fingerprints could evaluate objectively the quality of herbal medicines.

In order to assess and compare the activity of the components in the Dihuang and Shu Dihuang samples, verbascoside was used as a positive control to obtain the relative activity of the ABTS^+•^ inhibition peaks. The results found that the active components in the two herbs were markedly different. Contents of some components in Dihuang were changed after being processed so as to induce their different activity in the two herbs. This might imply the different therapeutic effects of the two herbs in clinical to some extent. Thus, the “quantity-effect” research method displayed its important action in the quality evaluation and identification of the active compounds of herbal medicines.

## Conclusion

In this study, the on-line HPLC-UV-ABTS method and a “quantity-effect” research idea were developed to evaluate the quality and antioxidant activity of the Dihuang and Shu Dihuang samples from the different manufacturers. The results revealed that the antioxidants in the Shu Dihuang samples were obviously different from those in the Dihuang samples owing to the contents of some components having changed after Dihuang was processed. Iridoid glycosides were the main antioxidants and their contributions to the total activity were higher, while the new produced ingredients in Shu Dihuang displaying significant ABTS^+•^ inhibition activity could not be ignored. In short, the HPLC-UV-ABTS method was simple, rapid and reliable and could be applied widely to screen the antioxidants and evaluate the antioxidant activity of the complex extracts. The combination method of the chromatographic and active fingerprints could evaluate integratedly the active components of the complex matrixes and might be valuable and meaningful for improving the quality control of herbal medicines.

## Supplementary information

**Additional file 1.** A table for manufacturer source of the Dihuang and Shu Dihuang samples.

**Additional file 2.** The raw data for the functional equation of verbascoside as a positive control, negative peak area (x), concentration (y).

**Additional file 3.** The peak areas and potencies of the negative peaks in the thirteen batches of Dihuang and Shu Dihuang samples. Potency of each negative peak calculated by the functional equation of verbascoside.

**Additional file 4.** The raw data of Figs. 4 and 5. The potencies of 18 negative peaks in 13 batches of Dihuang and Shu Dihuang samples.

**Additional file 5.** The potencies of the negative peaks in the active fingerprints and their similarity evaluation. The similarity values of the active fingerprints of 13 batches of Dihuang and Shu Dihuang samples calculated by the angle cosine formula.

**Additional file 6.** The peak areas of the chromatoghraphic fingerprints and their similarity evaluation. The similarity values of the chromatographic fingerprints of 13 batches of Dihuang and Shu Dihuang samples calculated by the angle cosine formula.

## Data Availability

The datasets used and/or analyzed during the current study are available from the corresponding author on reasonable request.

## Abbreviations

ABTS: 2, 2′-azino-bis(3-ethylbenzothiazoline-6-sulfonic acid)
BCD: Biological chemistry detection
Ch. P.: Chinese Pharmacopoeia
DPPH: 2,2-diphenyl-1-picrylhydrazyl hydrate
HPLC: high performance liquid chromatographic
HPLC-FTMS: HPLC-fourier-transform mass spectrometry
HPLC-PDA: HPLC-photo-diode array
HPLC-UV-ABTS: high performance liquid chromatographic-ultraviolet detection-ABTS

## References

[1] Xu ZJ, Shu S, Li ZJ, Liu YM, Zhang RY, Zhang Y (2017). Liuwei Dihuang pill treats diabetic nephropathy in rats by inhibiting of TGF-β/SMADS, MAPK, and NF-kB and upregulating expression of cytoglobin in renal tissues. Medicine (Baltimore).

[2] Zhao M, Tao J, Qian D, Liu P, Shang E, Jiang S (2016). Simultaneous determination of loganin, morroniside, catalpol andacteoside in normal and chronic kidney disease rat plasma by UPLC-MS for investigating the pharmacokinetics of *Rehmannia glutinosa* and *Cornus officinalis* Sieb drug pair extract. J Chromatogr B.

[3] Yuhong Y, Qian L, Yu L, Yingqiang Z, Yanfen L, Shujing Y (2013). An n-of-1 trial service in clinical practice: testing the effectiveness of Liuwei Dihuang Decoction for kidney-yin deficiency syndrome. Evid Based Complement Alternat Med.

[4] Zhu H, Wang Y, Liu Z, Wang J, Wan D, Feng S (2016). Antidiabetic and antioxidant effects of catalpol extracted from *Rehmannia glutinosa* (Di Huang) on rat diabetes induced by streptozotocin and high-fat, high-sugar feed. Chin Med.

[5] Li ZF, He CL, Wang Y, Li MJ, Dai YJ, Wang T (2016). Enhancement of trichothecene mycotoxins of Fusarium oxysporum by ferulic acid aggravates oxidative damage in *Rehmannia glutinosa* Libosch. Sci Rep.

[6] Yu HH, Seo SJ, Kim YH, Lee HY, Park RK, So HS (2006). Protective effect of *Rehmannia glutinosa* on the cisplatin-induced damage of HEI-OC1 auditory cells through scavenging free radicals. J Ethnopharmacol.

[7] Chao JC-J, Chiang SW, Wang CC, Tsai YH, Wu MS (2006). Hot water-extracted Lycium barbarum and *Rehmannia glutinosa* inhibit proliferation and induce apoptosis of hepatocellular carcinoma cells. World J Gastroenterol.

[8] Liu CL, Cheng L, Ko CH, Wong CW, Cheng WH, Cheung DW (2012). Bioassay-guide disolation of anti-inflammatory components from the root of *Rehmannia glutinosa* and its underlying mechanism via inhibition of iNOS pathway. J Ethnopharmacol.

[9] Huang Y, Qin T, Huang Y, Liu Z, Bo R, Hu Y (2016). *Rehmannia glutinosa* polysaccharide liposome as a novel strategy for stimulating an efficient immune response and their effects on dendritic cells. Int J Nanomedicine.

[10] Feng WS, Li M, Zheng XK, Zhang N, Song K, Wang JC (2015). Two new ionone glycosides from the roots of *Rehmannia glutinosa* Libosch. Nat Prod Res.

[11] Lee SY, Kim JS, Chio RJ, Kim YS, Lee JH, Kang SS (2011). A new polyoxygenated triterpene and two new aeginetic acid quinovosides from the roots of *Rehmannia glutinosa*. Chem Pharm Bull.

[12] Song Q, Zhao Y, Zhang N, Zhang Q, Liu Y, Li J (2016). Establishment of HPLC fingerprint of *Rehmanniae radix* and its HPLC-ESI-MS analysis. Chin Tradit Herbal Drugs.

[13] Chinese Pharmacopoeia Commission (2015). Pharmacopoeia of the People’s Republic of China.

[14] Martínez-Cayuela M (1995). Oxygen free radicals and human disease. Biochimie.

[15] Lee KJ, Oh YC, Cho WK, Ma JY (2015). Antioxidant and anti-inflammatory activity determination of one hundred kinds of pure chemical compounds using offline and online screening HPLC assay. Evid Based Complement Alternat Med.

[16] Karaçelik AA, Küçük M, İskefiyeli Z, Aydemir S, De Smet S, Miserez B (2015). Antioxidant components of *Viburnum opulus* L. determined by on-line HPLC-UV-ABTS radical scavenging and LC-UV-ESI-MS methods. Food Chem.

[17] Niederländer HAG, van Beek TA, Bartasiute A, Koleva II (2008). Antioxidant activity assays on-line with liquid chromatography. J Chromatogr A.

[18] Liu J, Tian J, Li J, Azietaku JT, Zhang B, Gao X, Chang Y (2016). In-capillary DPPH-capillary electrophoresis-the diode array detector combined with reversed-electrode polarity stacking mode for screening and quantifying major antioxidants in Cuscuta chinensis lam. Electrophoresis.

[19] Ma H, Li J, An M, Gao XM, Chang YX (2018). A powerful on line ABTS-CE-DAD method to screen and quantify major antioxidants for quality control of Shuxuening Injection. Sci Rep.

[20] Peng WB, Zeng QH, Li DP, Ding TM, Tan JL, Ding XP (2016). Multiple on-line HPLC coupled with biochemical detection methods to evaluate bioactive compounds in Danshen injection. Biomed Chromatogr.

[21] Wang LX, Xiao HB, Liang XM, Bi KS (2002). Vectorial angle method for evaluating the similarity between two chromatographic fingerprints of Chinese herb. Yao Xue Xue Bao.

[22] Ding XP, Qi J, Chang YX, Mu LL, Zhu DN, Yu BY (2009). Quality control of flavonoids in *Ginkgo biloba* leaves by high-performance liquid chromatography with diode array detection and on-line radical scavenging activity detection. J Chromatogr A.

[23] Ding XP, Wang XT, Chen LL, Qi J, Xu T, Yu BY (2010). Quality and antioxidant activity detection of *Crataegus* leaves using high-performance liquid chromatography with diode array detector coupled to chemiluminescence detection. Food Chem.

[24] Shen HD, Fang JJ, Guo PC, Ding TM, Liu JF, Ding XP (2018). Study of anti-oxidants of *Rehmanniae Radix* and *Rehmannia Radix Praeparata* by HPLC-UV-DPPH method. Chin Tradit Herbal Drugs.

