# Supplementary material for: “Quantity-effect” research strategy for comparison of antioxidant activity and quality of Rehmanniae Radix and Rehmannia Radix Praeparata by on-line HPLC-UV-ABTS assay
Source: BMC Complement Med Ther. 2020 Jan 17;20:16. doi: 10.1186/s12906-019-2798-8 (PMC7076824; doi:10.1186/s12906-019-2798-8)
Supplement: Supplementary file 1 — Additional file 1. A table for manufacturer source of the Dihuang and Shu Dihuang samples. [file 12906_2019_2798_MOESM1_ESM.docx]

Table Identification of active components in Dihuang and Shu Dihuang sample by HPLC-FTMS

| Dihuang | Herb resource | Shu Dihuang | Herb resource | Manufacturer |
| --- | --- | --- | --- | --- |
| S01 | Hebei | P01 | Hebei | Hubei Jingui |
| S02 | Henan | P02 | Henan | Hubei Jingui |
| S03 | Henan | P03 | Henan | Wuhan Shencao |
| S04 | Henan | P04 | Hebei | Hubei Changjiangyuan |
| S05 | Henan | P05 | Henan | Hubei Lishizhen |
| S06 | Shanxi | P06 | Shanxi | Hubei Lishizhen |
| S07 | Henan | P07 | Henan | Bozhou Li’er |
| S08 | Henan | P08 | Henan | Aanhui Huangtai |
| S09 | Henan | P09 | Henan | Aanhui Sanzhen |
| S10 | Henan | P10 | Henan | Guangzhou Jidi’ao |
| S11 | Henan |  | ~~Henan~~ | Hangzhou Huadong |
|  | ~~Henan~~ | P11 | Henan | Zhejiang Chinese medicinal University |
| S12 | Henan | P12 | Henan | Hubei Hong’en |
| S13 | Henan | P13 | Henan | Anhui Yiyuantang |
